# Supplementary material for: Knowledge and Attitude of Iranian University Students toward Genital Warts
Source: Interdiscip Perspect Infect Dis. 2022 Jul 13;2022:6730476. doi: 10.1155/2022/6730476 (PMC11401711; doi:10.1155/2022/6730476)
Supplement: Supplementary Materials — The translated version of the Persian questionnaire was used in this study. [file 6730476.f1.pdf]

1. Demographic data
    - Age
    - Gender
    - Education level
    - Study field
    - Marietal status
    - Number of children

2. General knowledge about HPV
    - Have you ever heard about HPV?
    - Is it the causal agent for genital wart?
    - Is it a STD?
    - Is it the causal agent of servical cancer?
    - Can it be asymptomatic?
    - Can it be diagnosable with pop smear?
    - Is it curable?
    - Is it need to be treated immediately?
    - Is it treatable with antibiotics?
    - Is it detected in 90% of patients with servical cancer?0
    -
3. Which of following items are considered as genital wart risk factors:
    - Smoking
    - UV
    - OCP
    - Multi partnership
    - Alcohol
    - Intercourse in lower age
    - Diet
    - Genetic
    - Marietal status
4. Which of following factors are considered as transmission path or redisposing factor?
    - Kissing
    - Intercourse
    - Oral sex
    - Contact with warts on other areas of body
    - Shared toilet
    - Body fluids (semen, blood)
    - Shared underwear
    - Skin to skin contact in genital area
5. Others
    - can AIDS be caused from HPV?
    - Can genital herpes be caused from HPV?

- Can HPV cause serious health issues in men?
- Can HPV cause serious health issues in women?
- Can HPV infection be prevented by using condom?
- Can HPV cause infertility?

- Do you know about the fact that about half of sexually active adults are HPV-infected?

6. Students’ attitude towards HPV-infected ones:
    - Their freedom should be limited
    - They should be embarrassed
    - They can take care of children without any risk
    - I do not scare from meeting this people
    - They are dirty
    - I do not want to be friend of such people
    - They should be isolated in the society
